# Volume of mural thrombus plays a role in the elevation of inflammatory markers after endovascular aortic repair

**DOI:** 10.1186/s13019-018-0712-y

**Published:** 2018-04-12

**Authors:** Jae Hang Lee, Jin-Ho Choi, Eung-Joong Kim

**Affiliations:** Department of thoracic and cardiovascular surgery, Dongguk University Ilsan Hospital, Goyang, Gyeonggi South Korea

**Keywords:** Aortic aneurysm, Endovascular aortic repair, Inflammation, Thrombus

## Abstract

**Background:**

Although systemic inflammatory responses are common after endovascular aortic repair (EVAR), its etiology remains uncertain. It is normally well tolerated and has a benign course. This study was undertaken to investigate the possible etiology of post-EVAR inflammation by measuring volumes of chronic mural thrombus and fresh thrombus.

**Methods:**

The subjects of this study included 34 patients who underwent EVAR from February 2012 to July 2017. Inflammatory markers in all the patients were evaluated before surgery, using the highest value among the laboratory data up to 5 days after surgery, and postoperative computed tomographic angiography (CTA) was taken for all of them before their discharging. Volumes of mural thrombus and fresh thrombus were calculated by CTA. The mean interval from surgery to immediate postoperative CTA was estimated as 6.8 ± 4.0 days.

**Results:**

After undergoing EVAR, white blood cell (WBC) (*p* < 0.01), C-reactive protein (CRP) (p < 0.01) and erythrocyte sedimentation rate (ESR) (*p* = 0.01) were significantly elevated. Two groups were defined according to the post-implantation syndrome (PIS) by the criteria of systemic inflammatory response syndrome (SIRS);no significant differences were observed in any factors between the two groups. Classification of two groups by the criteria of increasing WBC and CRP revealed that inflammatory markers were significantly enhanced as the volume of mural thrombus increased (*p* = 0.03). However, no significant risk factor was found in view of aneurysmal growth after EVAR.

**Conclusion:**

Volume of mural thrombus is an important risk factor for the elevation of inflammatory markers after EVAR.

## Background

It is relatively common for a systemic inflammatory response to occur after endovascular aortic repair (EVAR). This phenomenon, known as post-implantation syndrome (PIS), was first described in 1999 [1]. PIS is defined when fever and leukocytosis occur in the absence of any suspected infections after undergoing EVAR; its incidence has been reported from 14 to 60% [2–4]. PIS is known to be transient, self-limiting and benign, but current the methods and needs of management are controversial.

The hypotheses of the etiologies for the development of PIS are varied, and the typical presumed causes are foreign body reaction and thrombus formation. Of these, there is a general consensus regarding the role of materials on PIS. Numerous studies showed that PIS occurred in endografts made of polyester rather than expanded polytetrafluoroethylene (ePTFE) [2, 5, 6]. Conversely, the relation with thrombus formation remains uncertain. Few study outcomes reportedly measured the volumes of mural thrombus and fresh thrombus for comparison.

This study aims to investigate the probable causative factors of PIS including thrombus volume in aneurysm, and to understand the relationship with aortic aneurismal growth after EVAR.

## Methods

### Study design and patient population

A single-center, retrospective, observational study was undertaken to review the medical records of patients. A total of 61 patients underwent aortic intervention at this hospital from February 2012 to July 2017. Of these, 34 patients were included as the study subjects, whereas exclusions comprised of thoracic endovascular aortic repair (TEVAR) (*n* = 24), ruptured abdominal aortic aneurysm (AAA) (n = 2), and infected AAA (*n* = 1).

### Variables of interest

Demographics (age, gender), risk factors (hypertension, diabetes mellitus, coronary artery occlusive disease, cerebrovascular accident, chronic renal failure), preoperative medications (antiplatelet agent, statin), computed tomographic findings (maximum aneurysm diameter, volume of fresh and pre-existing mural thrombus, gas formaiton) were checked for each patient. Clinical course (mortality and morbidity, intensive care unit (ICU) and hospital stay, the occurrence of PIS) and laboratory findings (white blood cell (WBC), platelet (PLT), C-reactive protein (CRP), and erythrocyte sedimentation rate (ESR)) were also recorded for each patients. The study patients were divided into two groups with regard to the various factors (PIS versus non-PIS, low WBC versus high WBC, low CRP versus high CRP, decreased AAA versus no change or increased AAA).

### Perioperative procedure

All the patients underwent EVAR under general anesthesia. Prophylactic antibiotics (1st generation cephalosporin) were administered 30 min before incision, along with intravenous heparin (body weight (kg) × 100 unit). Surgical approach included two inguinal incisions performed without pre-closing device. After exposure of both femoral arteries, purse-string suture was performed using 5–0 Prolene, according to the Seldinger technique. Endurant (Medtronic Inc., Santa Rosa, CA, USA) endograft made of polyester was used for the stent graft. Patients were administered aspirin (100 mg) from the day after surgery. All the surgeries were performed by a single surgeon.

### Blood samples and volume measurement

**Fig. 1:**
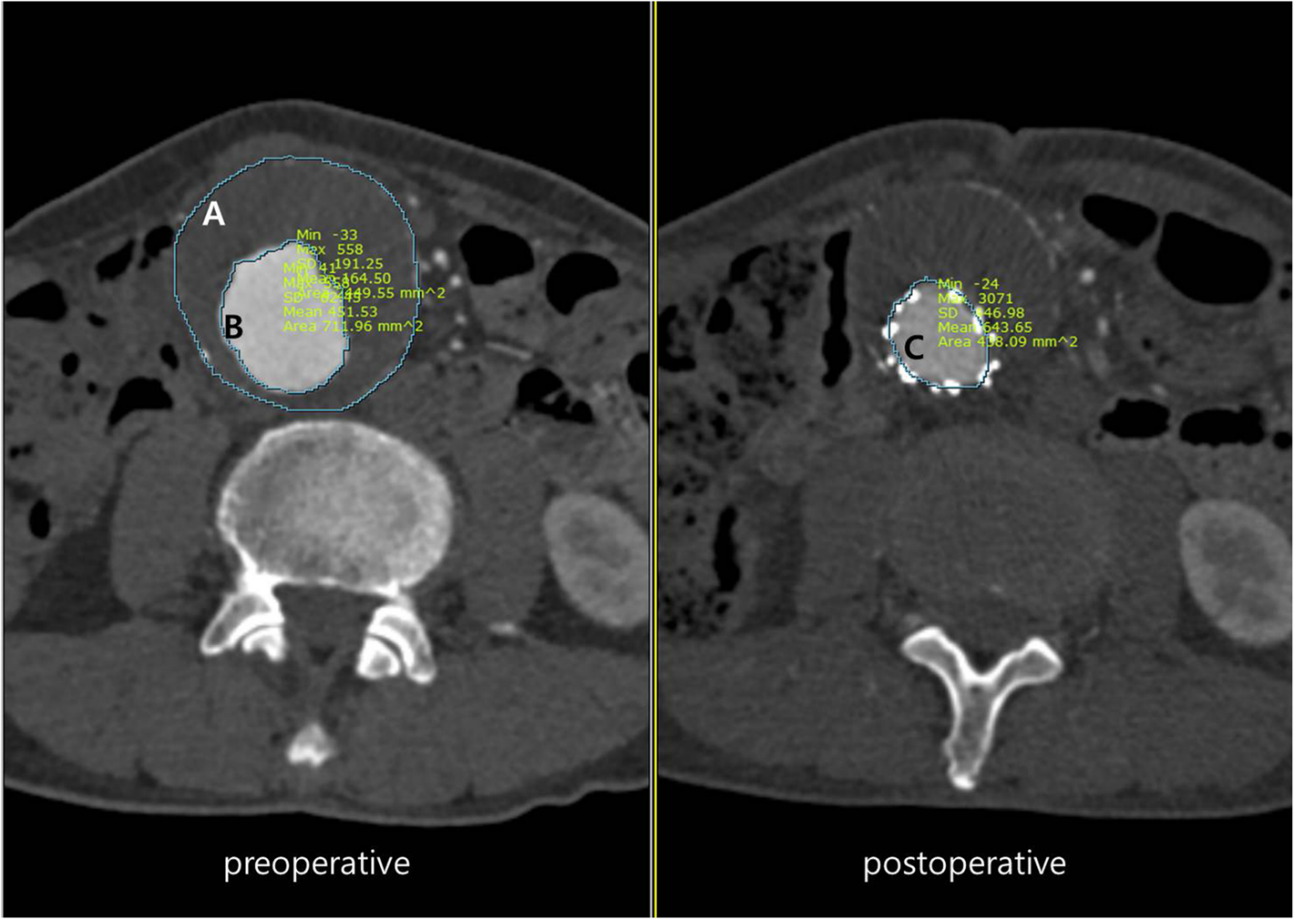
Volume measurement: Volume of mural thrombus was measured that subtracting **b** from **a** followed by multiplying by thickness. Volume of fresh thrombus was measured that subtracting **c** from **b** followed by multiplying by thickness

Inflammatory markers were evaluated in all patients before surgery, and laboratory data were used up to 5 days after the surgery with the highest value. All patients underwent postoperative computed tomographic angiography (CTA). The volume measure was calculated by assessing the area in axial view and multiplying by thickness. When the aneurysm extends to the iliac arteries, the volume of each iliac arteries were measured and added with the abdominal aortic aneurysmal sac. Mural thrombus was measured by preoperative CTA. The volume is measured by first obtaining the volume of the entire aneurysmal sac, and then subtracting the area filing the contrast. The volume of fresh thrombus was calculated by the volume of contrast filling in preoperative CTA and subtracting the graft volume observed in the immediate postoperative CTA (Fig. 1).

Mean interval from surgery to immediate postoperative CTA was 6.8 ± 4.0 days. PIS was defined as 1) fever over 38.0 °C and 2) leukocytosis, assessed at ≥ 12,000 white blood cell (WBC). These values were obtained as per the definition of systemic inflammatory response syndrome (SIRS).

### Statistical analysis

Continuous variables were expressed as mean ± standard deviation, whereas dichotomous variables were presented as counts and percentages. Comparisons of continuous variables were performed by Student t test for normally distributed variables, and Mann-Whitney U test for abnormally distributed variables. Chi-square test was used for categorical variables. All statistical analyses were performed using SPSS for Windows version 19.0 (SPSS Inc., Chicago, IL, USA), and statistical significance was considered for differences of 0.05 (*p* < 0.05).

## Results

Patient characteristics and peri-operative clinical data are presented in Table 1. There were no incidences of perioperative mortality or any major complications. Type 2 and type 1 endoleaks were observed in 4 and 1 patient, respectively. No open conversion was performed, and 2 patients underwent thrombectomy and stent insertion the day after surgery due to postoperative limb occlusion. CTA was performed for all patients before discharge, at postoperative day 6.8 ± 4.0.

**Table 1:** Baseline characteristics and peri-operative clinical data of patients

Patients’ characteristics	Number of patients (*n* = 34)
Age, years	75.7 ± 8.5
Male gender, n (%)	25 (74)
HTN, n (%)	25 (74)
DM, n (%)	5 (15)
CAOD, n (%)	6 (18)
CVA, n (%)	7 (21)
CRF, n (%)	2 (6)
Antiplatelet agent, n (%)	19 (56)
Statin, n (%)	16 (47)
Diameter of AAA, mm	57.2 ± 9.4
ICU stay, hours	22.4 ± 14.2
Hospital stay, days	9.9 ± 12.6

*HTN*: Hypertension; *DM*: Diabetes mellitus; *CAOD*: Coronary artery occlusive disease; *CVA*: Cerebrovascular accident; *CRF*: Chronic renal failure; *AAA*: Abdominal aortic aneurysm; *ICU*: Intensive care unit

**Fig. 2:**
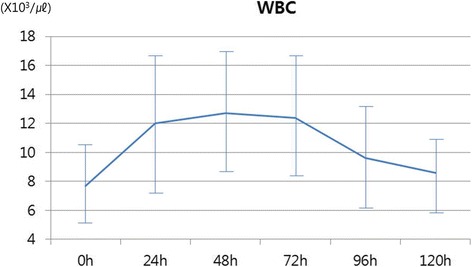
Distribution of white blood cell (WBC) count

**Fig. 3:**
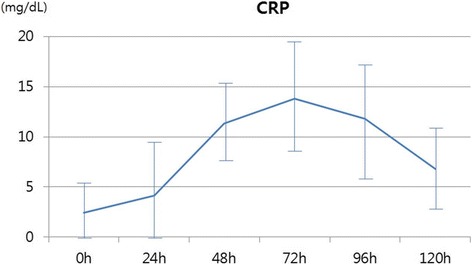
Distribution of C-reactive protein (CRP)

Postoperative elevations of inflammatory markers are presented in Table 2. There were no changes in the platelet counts; however, significant elevations of white blood cell (WBC), C-reactive protein (CRP), and erythrocyte sedimentation rate (ESR) were observed. Although they were not statistically evaluated, increasing trends were observed for WBC at postoperative 1–2 days, and for CRP at postoperative 3–4 days (Figs. 2 and 3).

**Table 2:** Preoperative and postoperative laboratory data

Variable	Preoperative value	peak postoperative value	*p*
WBC, × 10^3^/μl	7.8 ± 3.1	13.7 ± 4.6	< 0.01
PLT, ×10^3^/μl	226.2 ± 61.8	226.9 ± 6.5	0.93
CRP, mg/dL	2.5 ± 3.1	11.6 ± 5.4	< 0.01
ESR, mm/h	34.1 ± 31.6	83.1 ± 29.9	0.01

*WBC*: White blood cell; *PLT*: Platelet; *CRP*: C-reactive protein; *ESR*: Erythrocyte sedimentation rate

Table 3 shows the comparative results of two groups based on the definition of PIS. According to this, only WBC and fever showed statistical significance, other characteristics and CT findings did not show significant difference between the two groups. In addition, no statistically significant difference was found between the two groups during the clinical course of the patients.

**Table 3:** Patient characteristics, risk factors, and clinical data according to the presence or absence of postimplantation syndrome (PIS) after endovascular aortic repair (EVAR)

	PIS, *n* = 8	Non-PIS, *n* = 26	*P*
Age, years	78.8 ± 8.5	74.8 ± 8.4	0.25
Male gender, n (%)	6 (75)	19 (73)	1.00
HTN, n (%)	5 (63)	20 (77)	0.65
DM, n (%)	1 (13)	4 (15)	1.00
CAOD, n (%)	1 (13)	5 (19)	1.00
CVA, n (%)	1 (13)	6 (23)	1.00
CRF, n (%)	0 (0)	2 (8)	1.00
Antiplatelet agent, n (%)	4 (50)	15 (58)	1.00
Statin, n (%)	2 (25)	14 (54)	0.23
Mural thrombus, ml	64.8 ± 50.2	81.6 ± 69.1	0.53
Fresh thrombus, ml	65.8 ± 56.5	35.7 ± 34.1	0.19
Gas formation, n (%)	5 (63)	12 (46)	0.69
Fever, n (%)	8 (100)	3 (12)	< 0.01
WBC, ×10^3^/μl	17.1 ± 3.6	12.7 ± 4.5	0.02
PLT, ×10^3^/μl	258.4 ± 64.4	217.2 ± 63.4	0.12
CRP, mg/dL	12.7 ± 3.1	10.4 ± 7.0	0.22
ESR, mm/h	84.8 ± 25.4	69.3 ± 28.8	0.29
ICU stay, hours	25.0 ± 17.1	21.6 ± 13.5	0.57
Hospital stay, days	10.8 ± 6.3	9.6 ± 14.0	0.82

*PIS*: Post-implantation syndrome; *HTN*: Hypertension; *DM*: Diabetes mellitus; *CAOD*: Coronary artery occlusive disease; *CVA*: Cerebrovascular accident; *CRF*: Chronic renal failure; *WBC*: White blood cell; *PLT*: Platelet; *CRP*: C-reactive protein; *ESR*: Erythrocyte sedimentation rate; *ICU*: Intensive care unit

However, differences were statistically significant when WBC and CRP were considered as the inflammatory markers (Table 4). WBC was elevated as the patient’s age increased, and volume of mural thrombus was also greater. CRP was elevated with larger volume of mural thrombus. In general, the elevated inflammatory markers did not occur in accordance to fresh thrombus, but was proportional to the volume of mural thrombus. However, these criteria did not extend the hospital and intensive care unit (ICU) stays.

**Table 4:** Comparison of patient characteristics, risk factors, and clinical data based on the elevation of inflammatory markers

	Low WBC (< 12.0 × 10^3^/μl), *n* = 12	High WBC < 12.0 × 10^3^/μl), *n* = 22	*p*	Low CRP (< 10.0 mg/dL), *n* = 17	High CRP (> 10.0 mg/dL), *n* = 17	*p*
Age, years	71.6 ± 5.9	77.9 ± 9.0	0.04	73.0 ± 6.6	78.4 ± 9.5	0.06
Male gender, n (%)	10 (83)	15 (68)	0.44	13 (76)	12 (71)	1.00
HTN, n (%)	10 (83)	15 (68)	0.44	14 (82)	11 (65)	0.44
DM, n (%)	1 (8)	4 (18)	0.64	3 (18)	2 (23)	0.44
CAOD, n (%)	1 (8)	5 (23)	0.40	4 (24)	2 (23)	0.66
CVA, n (%)	4 (33)	3 (14)	0.21	5 (29)	2 (23)	0.40
CRF, n (%)	1 (8)	1 (5)	1.00	2 (23)	0 (0)	0.49
Antiplatelet agent, n (%)	6 (50)	13 (59)	0.61	12 (71)	7 (41)	0.08
Statin, n (%)	6 (50)	10 (45)	0.80	10 (59)	6 (35)	0.17
Mural thrombus, ml	51.4 ± 3.2	92.0 ± 7.4	0.03	53.6 ± 6.5	101.7 ± 7.9	0.03
Fresh thrombus, ml	49.5 ± 4.4	39.1 ± 4.0	0.49	41.8 ± 3.9	43.8 ± 4.9	0.89
Gas formation, n (%)	6 (50)	11 (50)	1.00	7 (41)	10 (59)	0.30
ICU stay, hours	17.2 ± 9.0	25.3 ± 15.9	0.11	21.9 ± 15.8	23.0 ± 13.0	0.83
Hospital stay, days	6.7 ± 4.5	11.6 ± 15.1	0.28	11.1 ± 17.1	8.6 ± 5.3	0.58

*WBC*: White blood cell; CRP: C-reactive protein; *HTN*: Hypertension; *DM*: Diabetes mellitus; *CAOD*: Coronary artery occlusive disease; *CVA*: Cerebrovascular accident; *CRF*: Chronic renal failure; *ICU*: Intensive care unit

Lastly, we reviewed the risk factors of aneurysmal growth and compared them with the last follow up CTA. The interval from the surgery to the last follow up CTA was 12.5 ± 13.8 months. We observed a decrease in the aneurysm size in 22 of the 34 patients, and an increase in 4 patients after the EVAR. The remaining 8 patients did not show any changes in the aneurysm size. As seen in Table 5, no correlation was found between any of the characteristics, including thrombus volume and inflammatory markers, upon reviewing the two groups of decreased aneurysm size and non-decreased aneurysm size.

**Table 5:** Risk factor analysis for aneurysmal growth after endovascular aortic repair (EVAR)

	Decreased AAA, *n* = 22	No change or increased AAA, *n* = 12	*p*
Age, years	75.5 ± 9.2	76.1 ± 7.4	0.85
Male gender, n (%)	15 (68)	10 (83)	0.44
HTN, n (%)	15 (68)	10 (83)	0.44
DM, n (%)	5 (23)	0 (0)	0.14
CAOD, n (%)	4 (18)	2 (17)	1.00
CVA, n (%)	4 (18)	3 (25)	0.68
CRF, n (%)	1 (5)	1 (8)	1.00
Antiplatelet agent, n (%)	11 (50)	8 (67)	0.35
Statin, n (%)	9 (41)	7 (58)	0.33
Diameter of AAA, mm	56.7 ± 8.3	58.1 ± 11.5	0.70
Mural thrombus, ml	69.3 ± 42.6	93.0 ± 93.5	0.42
Fresh thrombus, ml	45.1 ± 40.3	38.4 ± 45.3	0.66
Gas formation, n (%)	10 (45)	7 (58)	0.47
Fever, n (%)	9 (41)	2 (17)	0.25
WBC, ×10^3^/μl	13.4 ± 4.5	14.3 ± 5.0	0.58
PLT, ×10^3^/μl	218.1 ± 70.0	243.1 ± 55.0	0.29
CRP, mg/dL	10.7 ± 6.0	11.5 ± 7.2	0.74
ESR, mm/h	80.0 ± 21.0	55.5 ± 37.9	0.07

*AAA*: Abdominal aortic aneurysm; *HTN*: Hypertension; *DM*: Diabetes mellitus; *CAOD*: Coronary artery occlusive disease; *CVA*: Cerebrovascular accident; *CRF*: Chronic renal failure; *WBC*: White blood cell; *PLT*: Platelet; *CRP*: C-reactive protein; *ESR*: Erythrocyte sedimentation rate

## Discussion

Since EVAR is a popular method for the treatment of abdominal aortic aneurysm, studies on PIS have been widely conducted. Its incidence varies due to the ambiguity of PIS definition. For example, some authors defined PIS with regards to fever and leukocytosis, while others consider fever and elevated CRP [2, 5]. In this study, PIS was defined according to the SIRS definition by considering fever and leukocytosis. However, inflammatory markers such as CRP are also considered to be strongly related to PIS. In fact, many investigators have studied not only on WBC, CRP, and PLT, but also on various types of inflammatory markers including interleukin 6 and 8 (IL-6, IL-8), tumor necrosis factor α (TNF-α), and interferon gamma (IFN-γ) [6–9]. Moreover, a study was also conducted using genetic tests such as messenger ribonucleic acid (RNA) and protein analyses [10]. The common conclusion of these studies was that EVAR causes a systemic inflammatory reaction.

The associations between PIS and patient outcomes have been well established in most studies. Although PIS causes prolonged hospitalization and difficulty in postoperative recovery, most of the cases are known to be benign and self-limiting [2, 5–7, 11, 12]. On the other hand, several reports have alerted severe complications [13–15]. The authors of these studies revealed that PIS was associated to adverse events such as pulmonary dysfunction, cardiovascular events, renal insufficiency, multisystem organ failure, etc. Especially, Arnaoutoglou et al. reported a significant number of adverse events including major cardiovascular events, renal failure, and death, being 25.9% in the PIS group and 2.9% in the non-PIS group at 30 days after surgery [6]. However, in this study, major complications were not reported for all the patients; moreover, hospital and ICU stays were not extended, which differed from the results of existing studies.

Numerous studies have also been conducted on the etiology of PIS. Some consistency was observed in the relationship between the materials of endograft and PIS. Most of the studies reported frequent occurrences of PIS in the stent graft made of polyester than that made of ePTFE [2, 5–7]. In particular, Arnaoutoglou et al. claimed polyester endograft a 10 times higher independent predictor of PIS. Another theory on the etiology of PIS is the possibility of bacterial translocation by transient colonic ischemia [16]. This may result from occlusion or microembolism in the inferior mesenteric artery (IMA) or internal iliac artery (IIA). In addition, although it is believed that the operation time and contrast volume could possibly contribute to the etiology, the evidences are not sufficient.

Lastly, the shape and nature of the aortic aneurysm needs to be considered. One of the remarkable examples was mural thrombus formation in the aneurysm on which few studies have been conducted. Investigations tried to correlate PIS only with the maximal diameter of aneurysm or thickness of mural thrombus, but no correlation was observed [17, 18]. Very few studies tried to investigate the correlation with PIS by calculating the volume of thrombus. One study by Kakisis et al. was similar to our current study. Their results concluded that new-onset fresh thrombus might cause PIS rather than chronic mural thrombus [19]. This is interestingly inconsistent with our study. Kakisis et al. tried to explain PIS that fever occurred by absorbing blood as in deep vein thrombosis (DVT) or pulmonary embolism. Yet, they have the limitation not to consider the bias of materials since they enrolled the mixed cases with endografts made of ePTFE and polyester. This differed from our study, which has the advantage to eliminate the bias of graft materials by using a single product for all patients. The results of our study could be consistent with those in the previous studies. Swartbol et al. observed high amounts of IL-6 in mural thrombus in their study, demonstrating that released IL-6 upon the endovascular procedure stimulated to elevate WBC count [20], thereby concluding similar to our study that volume of mural thrombus can be a cause of PIS.

There were several limitations in this study. First, the study had a retrospective study design and second, the relatively small number of patients enrolled. Third, the inflammatory markers were not checked diversely, and hence we were unable to get the results of laboratory data such as TNF-α or IL-6 and IL-8 as seen in other studies. Lastly, the duration of CTA follow up was too short to check aneurysmal growth. Thus, a large number of patients and long-term follow-up period are required for more complete results.

## Conclusions

From this study, we showed that the volume of pre-existing mural thrombus is an important risk factor for the elevation of inflammatory markers after EVAR.

## Abbreviations

AAA: Abdominal aortic aneurysm
CAOD: Coronary artery occlusive disease
CRF: Chronic renal failure
CRP: C-reactive protein
CTA: Computed tomographic angiography
CVA: Cerebrovascular accident
DM: Diabetes mellitus
DVT: Deep vein thrombosis
ePTFE: Expanded Polytetrafluoroethylene
ESR: Erythrocyte sedimentation rate
EVAR: Endovascular aortic repair
HTN: Hypertension
ICU: Intensive care unit
IFN: Interferon
IIA: Internal iliac artery
IL: Interleukin
IMA: Inferior mesenteric artery
PIS: Post-implantation syndrome
PLT: Platelet
RNA: Ribonucleic acid
SIRS: Systemic inflammatory response syndrome
TEVAR: Thoracic endovascular aortic repair
TNF: Tumor necrosis factor
WBC: White blood cell
